# Recombinant Expression of a Ready‐to‐Use EGF Variant Equipped With a Single Conjugation Site for Click‐Chemistry

**DOI:** 10.1002/elsc.70015

**Published:** 2025-03-17

**Authors:** Melanie Krass, Meike Kolster, José Ignacio Valenzuela, Lena Moldenhauer, Marten Kagelmacher, Nicole Niesler, Alexander Weng, Marino Zerial, Gregor Nagel, Hendrik Fuchs

**Affiliations:** ^1^ Institute of Diagnostic Laboratory Medicine, Clinical Chemistry and Pathobiochemistry Charité–Universitätsmedizin Berlin, Corporate member of Freie Universität Berlin and Humboldt‐Universität zu Berlin Berlin Germany; ^2^ Institut für Pharmazie Freie Universität Berlin Berlin Germany; ^3^ Max Planck Institute of Molecular Cell Biology and Genetics Dresden Germany; ^4^ Institut für Chemie und Biochemie Freie Universität Berlin Berlin Germany

**Keywords:** click‐chemistry, epidermal growth factor, ligand variant, single conjugation site, targeted therapy

## Abstract

The epidermal growth factor (EGF) receptor is commonly targeted in cancer therapy because it is overexpressed in many malignant cells. However, a general problem is to couple the targeting moieties and the drug molecules in a way that results in a homogeneous product. Here, we overcome this issue by engineering a variant of EGF with a single conjugation site for coupling virtually any payload. The recombinant EGF variant K‐EGF^RR^ was expressed in *E. coli* Rosetta with a 4–6 mg/L yield. To confirm the accessibility of the introduced functional group, the ligand was equipped with a sulfo‐cyanine dye with a loading of 0.65 dye per ligand, which enables tracking in vitro. The kinetics and affinity of ligand–receptor interaction were evaluated by enzyme‐linked immunosorbent assay and surface plasmon resonance. The affinity of K‐EGF^RR^ was slightly higher when compared to the wild‐type EGF (*K*_D_: 5.9 vs. 7.3 nM). Moreover, the ligand–receptor interaction and uptake in a cellular context were evaluated by flow cytometry and quantitative high‐content imaging. Importantly, by attaching heterobifunctional polyethylene glycol linkers, we allowed orthogonal click‐conjugation of the ligand to any payload of choice, making K‐EGF^RR^ an ideal candidate for targeted drug delivery.

**Abbreviations**
ADC: antibody‐drug conjugates
AE: ArcticExpress
APC: allophycocyanin
BCA: bicinchoninic acid
Cy3: cyanine 3
Cy5: cyanine 5
DAPI/CMB: 4',6‐Diamidino‐2‐phenylindole/CellMask Blue
DAR: drug–antibody ratio
DBCO: dibenzocyclooctyne
DMEM: Dulbecco's modified eagle medium
DNA: deoxyribonucleic acid
DOTA: dodecane tetraacetic acid
DPBS: Dulbecco's phosphate buffered saline
EDTA: ethylenediaminetetraacetic acid
EGF: epidermal growth factor
EGFR: epidermal growth factor receptor
ELISA: enzyme‐linked immunosorbent assay
FBS: fetal bovine serum
FSC: forward scatter
HBS‐EP: HEPES‐buffered saline with EDTA and polysorbate 20
HEPES: 4‐(2‐hydroxyethyl)‐1‐piperazineethanesulfonic acid
IPTG: isopropyl‐β‐D‐thiogalactopyranosid
*K*_D_
: equilibrium dissociation constant
LB: lysogeny broth
mAB: monoclonal antibody
NHS: N‐hydroxysuccinimide
NiNTA: nickel‐nitriloacetic acid
OD: optical density
PBS: phosphate‐buffered saline
PEG: polyethylene glycol
RNA: ribonucleic acid
SA: streptavidin
SDS‐PAGE: sodium dodecyl‐sulfate polyacrylamide gel electrophoresis
siRNA: small interfering RNA
SPAAC: strain‐promoted azide‐alkyne cycloaddition
SPR: surface plasmon resonance
TMB: 3,3′,5,5′‐tetramethylbenzidine
TRIS: tris(hydroxymethyl)aminomethane
UV/VIS: ultra violet/visible spectroscopy
wt: wild‐type

## 1 Introduction

Cancer is one of the leading causes of death worldwide. In 2020 alone, cancer was responsible for 10 million fatalities, according to the World Health Organization [1]. Conventional cancer treatment methods, such as surgery, radiation, and chemotherapy, have their limitations and disadvantages. Surgery's efficacy is often limited by the tumor's accessibility, while radiation and chemotherapy affect not only cancer cells but also healthy tissue, leading to severe adverse effects [2]. Therefore, there is a need for new and innovative treatment approaches. Targeted therapy inhibits molecular pathways that are crucial for tumor growth and maintenance, while immunotherapy seeks to stimulate a host immune response that results in permanent tumor destruction [3]. Both minimizes the adverse effects of undirected damage to off‐targeted cells and widens the therapeutic window, allowing for lower doses.

**Summary**

- This ready‐to‐use EGF variant enables easy and site‐specific coupling to any effector molecule—regardless of whether the effector is a protein—for targeted therapies aimed at cells that overexpress the EGF receptor on their surface.
- Drugs for targeting tumor cells with toxins of any kind or with small interfering RNA can be produced quickly and will result in very homogeneous coupling molecules.
- This will also accelerate screening of effector candidates.

The epidermal growth factor receptor (EGFR) is a well‐established target for treating cancer, as many malignancies overexpress this receptor, which plays an important role in cell growth and proliferation. Its abnormal activation can trigger cancer development [4]. EGFR undergoes endocytosis upon ligand binding, initiating conformational changes that lead to the formation of homo‐ or heterodimers with other receptors. This process activates the tyrosine kinase domain of EGFR and triggers phosphorylation of its C‐terminal tyrosine residues, thereby activating downstream signaling pathways [5, 6]. Therefore, monoclonal antibodies (mAbs) or tyrosine kinase inhibitors are applied to treat cancer because they specifically inhibit this receptor. Since the early 1980s, mAbs have been investigated as potential treatments in humans and been hailed as the prototypical magic bullet drug because of their inherent capacity for specificity [7]. Four anti‐EGFR‐mAbs (cetuximab, panitumumab, nimotuzumab, and necitumumab) are already on the market, and others are in clinical trials [8]. The mAbs competitively inhibit the EGFR by binding at the extracellular domain of the receptor, which prevents activation of its tyrosine kinase function. Tyrosine kinase inhibitors can inhibit both autophosphorylation and subsequent activation of downstream signaling [9, 10, 11]. Besides acting as direct therapeutic agents, mAbs can also serve as targeting moieties to actively deliver small chemotherapeutic toxins, macromolecules, or prodrug activation enzymes to target cells [12, 13, 14]. In addition to antibodies, natural or modified protein ligands (e.g., transferrin, interleukin‐2) can be used as targeting moieties for drug delivery [15, 16]. A well‐known ligand for EGFR is the EGF. It benefits from its compact size, which is significantly smaller than antibodies, and its natural evolutionary adaptation to the EGFR.

Although several antibody–drug conjugates (ADCs) are already approved by the U.S. Food and Drug Administration and the European Medicines Agency (e.g., Enhertu, Trodelvy, Besponsa) [17], they also exhibit a major drawback, namely lacking homogeneity. Even the approved ADCs constitute a mixture of different drug–antibody ratios (DAR) due to multiple conjugation sites on the mAb. Likewise, antibodies with identical DAR are heterogeneous because modification occurs at alternate positions. This heterogeneity can lead to different pharmacokinetics of the ADCs, interfere with receptor binding, and impair control over the precise dosage when only the average of DAR is known [18, 19, 20, 21]. Despite clinical trials with ADCs allowing for heterogeneity, there is currently a strong need for methods to produce more consistent and uniform products. One such method for mAbs is to introduce unnatural amino acids into the backbone or to undergo glycosyl remodeling [22, 23].

So far, EGF has been expressed in eukaryotic systems as well as in *E. coli* [24, 25, 26]. Due to its small size and three disulfide bonds, obtaining a correctly folded and soluble product during expression has proven to be quite challenging. Previous attempts to address this challenge, such as those by Su et al. [27] and Ma et al. [28], involved the incorporation of a small ubiquitin‐related modifier (SUMO). However, this strategy introduced an additional SUMO cleavage step using a protease.

Previous research has explored the replacement of the lysines and some arginines with neutral amino acids such as serine or glutamine (K28Q, R45S, K48S, and R53S) [29] or replacement of the lysines with two arginines (RR), but also with serin and arginine (SR, RS) [30]. In all cases, the binding affinity to EGFR was maintained. The RS and SR variants exhibited a comparable phosphorylation level of the extracellular signal‐regulated kinase (ERK) while the RR variant showed lower ERK phosphorylation than the wild‐type. In both studies, the N‐terminal amino group was used for coupling reactions; however, irreversible addition of an acyl moiety to the terminal alpha amino group of a peptide chain can prevent conjugation [31, 32].

Here, we modify EGF for drug targeting in a way that it contains exactly one defined conjugation site for active compounds and that this conjugation site is bioorthogonal and does not interfere with receptor binding and cellular uptake. This next‐generation EGFR targeting technology will significantly improve the toolbox for targeted cancer therapy.

## 2 Materials and Methods

### 2.1 Recombinant Expression of K‐EGF^RR^ in *E. coli*

The recombinant plasmid pET11d‐K‐EGF^RR^ (custom synthesis by BioCat, Heidelberg, Germany) was introduced into the *E. coli* strains BL21(DE3), NiCo21(DE3), Rosetta(DE3), and ArcticExpress(DE3) (AE(DE3)) (all obtained from Agilent, Santa Clara, US). The expression protocol was adapted from wild‐type EGF expression methods described by Kim et al. [33] and Liu et al. [34] The transformed bacteria were plated on agar plates containing 50 µg/mL ampicillin and incubated (16 h, 37°C). Afterward, an individual colony was selected and transferred into 2 mL of Luria Bertani (LB) medium, supplemented with 50 µg/mL ampicillin for all *E. coli* strains. For Rosetta(DE3), an additional 25 µg/mL chloramphenicol was added, and for AE(DE3), 50 µg/mL of gentamycin was included, and the bacteria culture was incubated (6 h, 37°C, 200 rpm). Subsequently, 50 µL of the turbid bacteria solution was added to 100 mL LB medium (with 50 µg/mL ampicillin) and incubated (16 h, 37°C, 200 rpm). Then, 50 mL of the overnight culture were added to 800 mL LB medium (with 50 µg/mL ampicillin) or until the optical density at 600 nm (OD_600_) reached approx. 0.3 ± 0.1. The culture was further incubated to an OD_600_ of 0.8 ± 0.1, at which point expression was induced with isopropyl‐β‐D‐thiogalactopyranoside (IPTG, Carl Roth, Karlsruhe, Germany) following the manufacturer's instruction. The bacteria solution was then shaken (16 h, 4°C (for AE(DE3)); 3 h, 37°C (for the other strains); 200 rpm) and afterward centrifuged (4°C, 5000 × *g*, 20 min). The supernatant was discarded, and the pellet was reconstituted in 20 mL of Dulbecco's phosphate buffered saline (DPBS) (Lonza Group, Basel, Switzerland). This suspension was frozen for storage until needed, with a minimum overnight freezing period.

### 2.2 Purification of K‐EGF^RR^

The bacteria suspension underwent lysis using a French press (Thermo Electron Corporation, Waltham, USA) and was subsequently centrifuged (4°C, 30,000 × *g*, 30 min). K‐EGF^RR^ was found to be within inclusion bodies and had to be recovered by adopting the procedure of Patra et al. [35]. Therefore, the supernatant was discarded and the inclusion bodies in the pellet were resuspended two times in washing buffer A [50 mM tris(hydroxymethyl)aminomethane (TRIS) (Carl Roth), 5 mM ethylenediaminetetraacetic acid (EDTA) (Carl Roth), 0.1% Tween‐20 (Applichem, Darmstadt, Germany), pH 8.5], one time in washing buffer B (50 mM TRIS, pH 8.0) and one time in ultrapure water. After each resuspension, the solution underwent centrifugation (4°C, 30,000 × *g*, 20 min) and the supernatant was discarded. The pellet was dissolved in 20 mL solubilization buffer [50 mM TRIS, 2 M urea (Merck, Darmstadt, Germany), pH 12] and incubated for 30 min at room temperature. The protein solution was then dialyzed against a dialysis buffer [20 mM TRIS, 1 mM EDTA, 1 mM reduced glutathione (Sigma Aldrich, St. Louis, USA), 0.1 mM oxidized glutathione (Carl Roth, Karlsruhe, Germany), 10 wt% sucrose (Carl Roth), pH 8.5] overnight. Afterward, the solution containing K‐EGF^RR^ was purified via nickel‐nitrilotriacetic acid (NiNTA) affinity chromatography (Macherey‐Nagel, Düren, Germany) and fractionated by elution with imidazole (31, 62, 125, 250 mM) (Carl Roth), resulting in a yield of 250 µg K‐EGF^RR^ (for AE(DE3)) and 5–6 mg K‐EGF^RR^ (for Rosetta) per 1 L expression batch. The yield was determined via bicinchoninic acid (BCA) assay (Thermo Scientific, Waltham, USA) and UV/VIS absorption (SpectraMax 340 PC).

### 2.3 Conjugation of Dye and Click‐Adapter

10 equivalents of sulfo‐cyanine‐3‐N‐hydroxysuccinimid (sulfo‐Cy3‐NHS) or sulfo‐cyanine‐5‐NHS dye (Lumiprobe, Hannover, Germany) or linker (DBCO‐PEG_12_‐NHS, DBCO‐PEG_3_‐S‐S‐NHS) (Conju Probe, San Diego, USA) were added to the EGF (in PBS) and incubated (16 h, 25°C, 800 rpm). Afterward, the protein solution was purified via PD10 columns (Cytiva, Marlborough, USA) according to the manufacturer's protocol. The yield was again determined via BCA assay (Thermo Scientific, Waltham, USA) and UV/VIS absorption (SpectraMax 340 PC).

### 2.4 Flow Cytometry

MDA‐MB 468 (ATCC HTB‐132), A‐431 (ATCC CRL‐1555), and A2058 (ATCC CRL‐11147) cells were cultured in BioWhittaker Dulbecco's Modified Eagle's Medium (DMEM, 4.5 g glucose/L) (Lonza) supplemented with 10% fetal bovine serum (FBS) (Bio&SELL GmbH, Nürnberg, Germany) in a humidified 5% CO_2_ atmosphere at 37°C. For internalization experiments, 20,000 cells were seeded in each well of a 96‐well plate in 100 µL of DMEM and incubated (24 h, 37°C). Afterward, the culture medium was entirely replaced with fresh medium containing the compounds to be evaluated. To assess concentration‐dependent uptake, cells were exposed to cyanine‐5 modified K‐EGF^RR^ (Cy5‐K‐EGF^RR^) solutions with varying concentrations for 24 h. For investigating time‐dependent uptake, a solution with a constant Cy5‐K‐EGF^RR^ concentration of 100 nM was added to the cells at different time points within the incubation period. To evaluate the competition between the ligand variant and its unlabeled counterpart, cells were incubated for 4 h with a constant concentration of Cy5‐K‐EGF^RR^ (100 nM) and varying concentrations of nonlabeled K‐EGF^RR^.

After incubation, the cells were washed twice with 150 µL of DPBS (Lonza) to analyze the in vitro internalization studies. Subsequently, 150 µL of trypsin/versene (Lonza) solution was applied to detach the cells. The resulting cell suspensions were then transferred to microcentrifuge tubes and kept on ice until ready for fluorescence cytometry analysis using a CytoFLEX flow cytometer (Beckmann Coulter, Indianapolis, USA). Blank cell suspensions were used to manually establish the gating of single cells based on their forward scatter (FSC‐height vs. FSC‐width). A minimum of 10,000 single cells were included in the analysis of each condition. To assess the Cy5‐related fluorescence signal, the signal detected in the allophycocyanin (APC)‐channel (excitation: red laser, 638 nm, emission bandpass filter: 660/10 nm) was evaluated.

### 2.5 Cellular Uptake of K‐EGF and Automated Imaging

HeLa Kyoto cells were cultured in DMEM supplemented with 10% FBS and 50 µg/mL gentamycin in a humidified 5% CO_2_ atmosphere at 37°C. The knockdowns were achieved through reverse transfection of siRNAs. Briefly, 5 µL of siRNA‐EGFR (Dharmacon, Lafeyette, USA), siRNA‐Eg5 (Ambion, Thermo Scientific, Waltham, USA), or siRNA‐lowGC (Invitrogen, Waltham, USA) and 5 µL interferin were added to a 384‐well plate and incubated for 15 min at 25°C. For the mock‐transfected control, no siRNA was added. Afterward, 250 cells in 40 µL were added to each well of the 384‐well plate and incubated for 72 h at 37°C. Then, the media was replaced with 20 µL FBS‐free medium. Wild‐type EGF directly labeled with 2–3 Alexa‐647/ligand (20 µL, 9 nM) or K‐EGF^RR^ labeled with 0.25 Sulfo‐Cy5‐NHS/ligand (20 µL, 9 nM) diluted in serum‐free medium were added to the cells and incubated at 37°C and 5% CO_2_ for varying time intervals. Then, the cells were washed two times using a HydroSpeed plate washer (Tecan, Männedorf, Swiss), followed by fixation with 3.7% formaldehyde (40 µL, 15 min, 25°C) and staining with 4',6‐diamidino‐2‐phenylindol/ CellMask Blue (DAPI/CMB). High‐magnification images were captured using an automated spinning disc confocal microscope (CellVoyagerTM 7000, Yokogawa, Marl, Germany) equipped with three Andor Neo 5.5 sCMOS cameras and a 40 × 0.95 ULPSAPO objective. CellMask Blue and DAPI were visualized using laser excitation at 405 nm and an emission bandpass filter BP445/45, while Alexa 647 and Cy5 were imaged with a 640 nm laser and a BP676/29 filter.

### 2.6 Image Analysis

Images were analyzed using the open‐source software CellProfiler [36]. Briefly, in corrected images from the CV7000 Yokogawa microscope, nuclei, cytoplasm, and EGF cargo spots were segmented and quantified in CellProfiler. Segmentation outlines, and images from both channels were combined to inspect the segmentation results manually. Object data from cells and their measurements were exported as result .csv files. The result files were loaded into the open source data analysis software KNIME [37], and the object data of cells per well (EGF‐dots per cell, EGF intensity, EGF‐dots intensity per cell, and number of cells) was aggregated as means. Customized R scripts were used for visualization, or the well's data tables were exported in different formats for further customized data processing and visualization.

### 2.7 Sandwich‐ELISA Assay

For the enzyme‐linked immunosorbent assay (ELISA), the wells of a streptavidin‐coated 96‐well‐plate (Thermo Scientific) were washed three times with 200 µL wash buffer (PBS, 0.05% Tween‐20 Detergent). Subsequently, 100 µL of the biotinylated EGF receptor (5 µg/mL; 500 ng/well) (Acro, Newark, USA) was added to each well. The plate was covered with an adhesive plastic seal and gently shaken on a rocking platform (16 h, 4°C). After the coating period, the solution was removed, and the wells were washed three times with 200 µL of the wash buffer. Afterward, 200 µL blocking solution (Diluent A (BioLegend, San Diego, USA), 2 h), 100 µL EGF (1 µg/mL; 100 ng/well; 1 h), 100 µL primary rabbit antibody (1:1000; anti‐EGF (16 h) (Abcam, Cambridge, UK), 100 µL secondary antibody (1:1000; goat anti‐rabbit immunoglobulins, 1 h) (Dako, Jena, Germany) and 100 µL 3,3′,5,5′‐tetramethyl‐benzidine (TMB) substrate (15 min) (Sigma Aldrich) were added. After each step, the wells were washed three times with 200 µL of the wash buffer. To stop the TMB reaction, 100 µL of 2 M sulfuric acid (Carl Roth) was added. The absorbance was measured at 450 nm via a spectrophotometer (SpectraMax 340 PC, Molecular Devices, San Jose, USA).

### 2.8 Surface Plasmon Resonance (SPR)

The interaction between the ligand and its corresponding receptor was investigated by SPR with a Biacore X100 SPR device using streptavidin‐coated sensor chips (SA Chip, Cytiva). The biotinylated EGFR was immobilized on the SA chip to reach a level of 5000 RU. This immobilization was carried out in HBS‐EP buffer [10 mM HEPES (Carl Roth) pH 7.4, 150 mM NaCl (Thermo Scientific), 3 mM EDTA, 0.005% v/v Surfactant P20 (Cytiva)] on flow cell 2 (Fc2), while the reference flow cell (Fc1) was left untreated. For the binding studies, EGF dissolved in HBS‐EP buffer was injected with a contact time of 180 s, followed by a dissociation time of 600 s. To regenerate the chip, it was treated with 10 mM NaOH (Carl Roth) with a contact time of 30 s and a stability time of 60 s. The equilibrium dissociation constant *K*_D_ was determined using the Biacore X100 Evaluation Software (version 2.0.2) and the steady state fit method.

## 3 Results

### 3.1 Design of an EGF Variant for Specific Targeting of Cancer Cells

**FIGURE 1:**
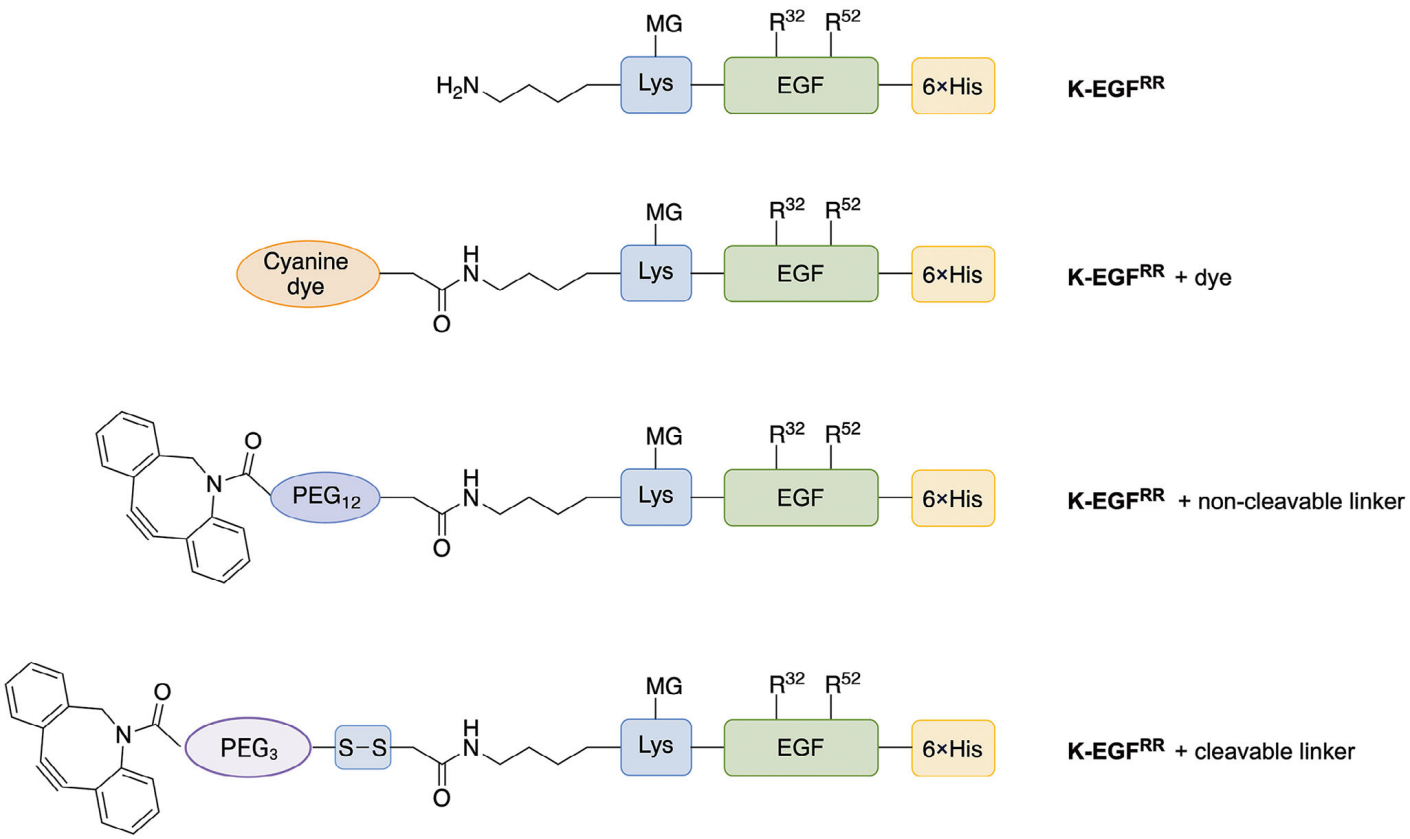
Design of the variant K‐EGF^RR^ with additional 6 × His‐Tag and its site‐specific modifications. R32 and R52 indicate the lysines, which were replaced by arginine (R) at positions 32 and 52. The artificially inserted lysine serves as a single conjugation site and is indicated in blue, whereas the His‐tag is depicted in yellow. The EGF variant was equipped with dye and non‐cleavable or cleavable PEG linkers by conjugating them at the single conjugation site. The schemes are not to scale.

EGFR is overexpressed in many malignant cells and targeted in clinically approved anticancer drugs. Therefore, we chose its natural ligand, EGF, as a cancer‐targeting moiety. We developed an EGF variant with only one single conjugation site to avoid cross‐linking or heterogeneity in the final product. In this variant, the two naturally occurring lysines at amino acid positions 32 and 52 within EGF were replaced with arginine (R) and an additional lysine (K) was added close to the N‐terminus for site‐specific conjugation (K‐EGF^RR^) (for sequences, see Figure S1), e.g., dyes or click‐adapters. Additionally, a 6 × His‐Tag was inserted at the C‐terminus for purification purposes (NiNTA) and was cloned into a pET11d vector (Figure 1).

### 3.2 Recombinant Expression and Purification of the EGF Variant K‐EGF^RR^

**FIGURE 2:**
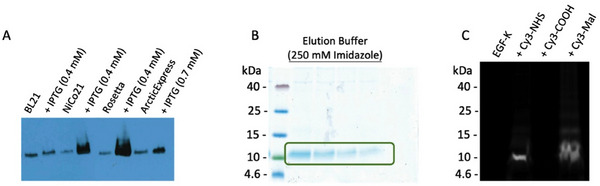
Successful expression leading to the pure K‐EGF^RR^. (A) Expression in different *E. coli* strains. (B) Elution of K‐EGF^RR^ expressed in Rosetta(DE3) after NiNTA affinity chromatography and (C) conjugation of Cy3‐dyes to K‐EGF^RR^ to confirm the accessibility of the functional groups.

Following the molecular design and cloning of the EGF variant, we determined the conditions for producing recombinant K‐EGF^RR^ with high yield and purity. To this end, the pET11d‐K‐EGF^RR^‐6 × His plasmid was transformed into different *E. coli* strains, namely BL21(DE3), NiCo21(DE3), Rosetta(DE3), AE(DE3) and treated with different amounts of IPTG. The expression level was highest in the case of NiCo21(DE3) and Rosetta(DE3) with IPTG activation (Figure 2A). However, purification by NiNTA yielded the desired and pure product only in the case of Rosetta(DE3) and AE(DE3). The elution fraction revealed the expected bands at approximately 7.6 kDa after sodium dodecyl sulfate polyacrylamide gel electrophoresis (SDS‐PAGE) (Figure 2B). In contrast to AE(DE3), where K‐EGF^RR^ was mainly recovered from the supernatant, the ligand in the Rosetta(DE3) strain was detected in the pellet indicating that the variant was completely encapsulated in inclusion bodies. Consequently, the ligand had to be released from them and refolded before purification using 2 M urea.

After purification, K‐EGF^RR^ was dialyzed against PBS. Due to its small size, K‐EGF^RR^ is more likely to interact with dialysis membranes. Therefore, low‐binding dialysis tubes were used to prevent product loss. The yield of pure K‐EGF^RR^ was successfully increased from 250 µg (AE(DE3)) to 5–6 mg (Rosetta(DE3)) per 1 L expression approach. To confirm the accessibility of the single artificial lysine and the eight naturally occurring cysteines, we treated the K‐EGF^RR^ with different dyes. To address the amino group of lysine, a cyanine‐3 dye with an *N*‐hydroxysuccinimide (NHS) ester (Cy3‐NHS) was used to form an amide bond. For the thiol group of the cysteine, on the other hand, a maleimide cyanine‐3 dye (Cy3‐Mal) was used to perform a thiol‐ene‐Michael addition. Additionally, K‐EGF^RR^ was treated with Cy3‐COOH as a negative control to exclude the unspecific interaction of dye and ligand with each other, as carboxylic acids should not be able to react with the functional groups at the ligand without activation. Accessibility was confirmed by SDS‐PAGE, where bands were observed in the Cy3 channel of a molecular imager in the case of Cy3‐NHS or Cy3‐Mal but not for Cy3‐COOH (Figure 2C). The appearance of a band when using Cy3‐Mal indicates that not all the disulfide bridges in EGF are closed in each molecule. Here, we have achieved the high‐yield production of pure K‐EGF^RR^, an EGF variant comprising a single conjugation site suitable for chemical functionalization.

### 3.3 K‐EGF^RR^ Dye and Click‐Adapter Functionalization for Live‐tracking and Orthogonal Payload Conjugation

**FIGURE 3:**
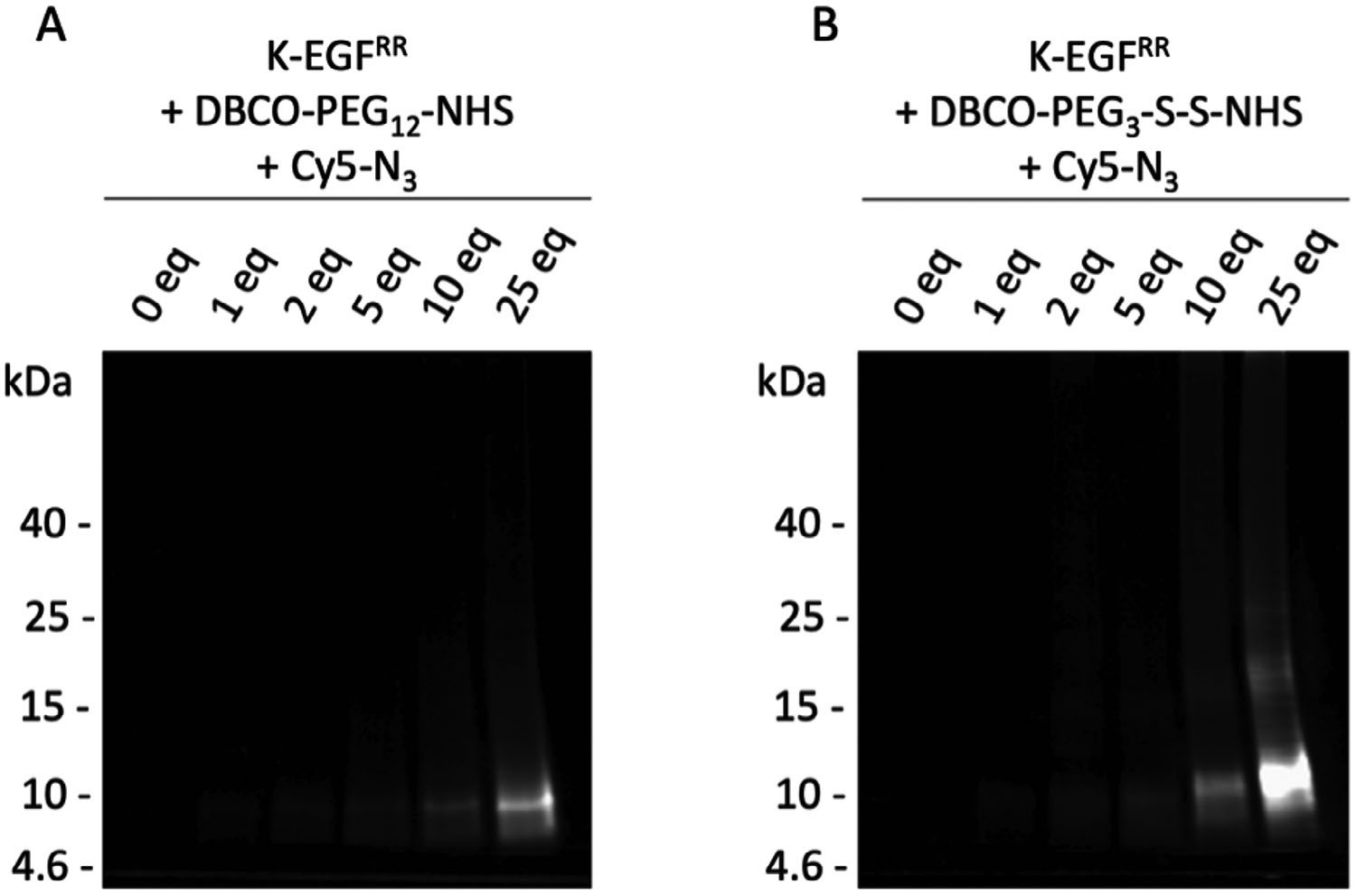
Successful conjugation of orthogonal linkers to K‐EGF^RR^. Conjugation of (A) non‐cleavable and (B) cleavable DBCO‐PEG‐NHS linker at K‐EGF^RR^ with different equivalents of the linker. Afterward, the K‐EGF^RR^‐linker samples were incubated with Cy5‐N_3_ to assess whether the linker was successfully conjugated. The images were obtained by using the Cy5‐channel of a VersaDoc device.

The highly efficient purification of K‐EGF^RR^ enables the creation of an EGFR‐targeting platform for efficient and reproducible conjugation of payloads by click‐chemistry. After proving the accessibility of the functional groups, the ligand K‐EGF^RR^ was equipped with dyes for the following in vitro studies and to determine the conjugation rate. Therefore, the single conjugation site of K‐EGF^RR^ was addressed, instead of the cysteines, to avoid the destruction of the ligand integrity. After treating the ligand with the dye, the amount per ligand was determined by UV/VIS‐spectroscopy by dividing the amount of dye by the amount of ligand. For the first experiments, Cy3‐NHS was used, but a fraction of free dye was detected in the purified Cy3‐K‐EGF^RR^ by SDS‐PAGE, which could interfere with the in vitro evaluations. For this reason, we switched to sulfo‐Cy5‐NHS, which is much less susceptible to aggregation under aqueous conditions due to its hydrophilicity and water solubility. The dye loading using sulfo‐Cy5‐NHS was 0.65 dye/ligand. To create a conjugation site for a drug or payload via click‐chemistry, the strain‐promoted azide–alkyne cycloaddition (SPAAC), a dibenzocyclooctyne (DBCO) group was introduced using a heterobifunctional polyethylene glycol (PEG) linker. The PEG linker provided an NHS ester for conjugation at the single lysine. In addition to a non‐cleavable linker (DBCO‐PEG_12_‐NHS), a cleavable linker (DBCO‐PEG_3_‐S‐S‐NHS) was chosen to introduce a redox‐labile disulfide bond that allows separation of the payload and the ligand after cellular internalization, which might be desirable for maximizing the activity of drugs. Because the UV/VIS spectroscopy could not accurately determine the quantity of linker per ligand due to their low concentrations, we verified the successful conjugation using SDS‐PAGE. For this purpose, we treated K‐EGF^RR^ equipped with the DBCO linker with Cy5‐azide, as the azide selectively reacts with the DBCO component of the linker. In the Cy5 channel, a distinct band at approximately 7.6 kDa is observed for each sample treated with varying equivalents of the linker (Figure 3).

The intensity of the K‐EGF^RR^ band increased in proportion to the number of linker equivalents used during the conjugation process. As expected, no band was detected in the Cy5 channel for the negative control, where Cy5‐azide was added to the K‐EGF^RR^ without a linker (see Figure 3, lanes “0 eq.”). This absence of a band in the negative control indicates that the band in the samples treated with the linker results from the successful conjugation and is not a consequence of nonspecific interactions between the dye and the ligand.

### 3.4 K‐EGF^RR^ Displays High‐Affinity Ligand–Receptor Interaction

**FIGURE 4:**
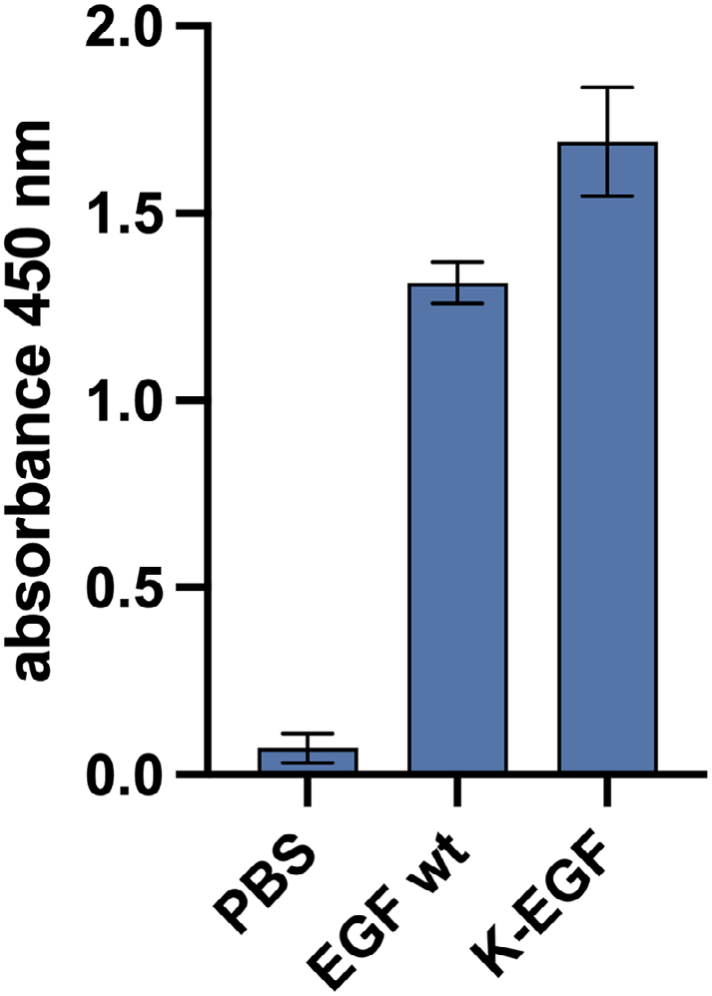
Higher EGFR binding affinity of K‐EGF^RR^ than commercially available wild‐type EGF determined by ELISA. Biotinylated EGFR was immobilized on streptavidin‐coated well plates and treated with K‐EGF^RR^/EGF. Absorbance was measured at 450 nm. The mean value ± SEM is shown (*N* = 3, *n* = 3; *N*: number of biological replicates, *n*: number of technical replicates per biological replicate).

**FIGURE 5:**
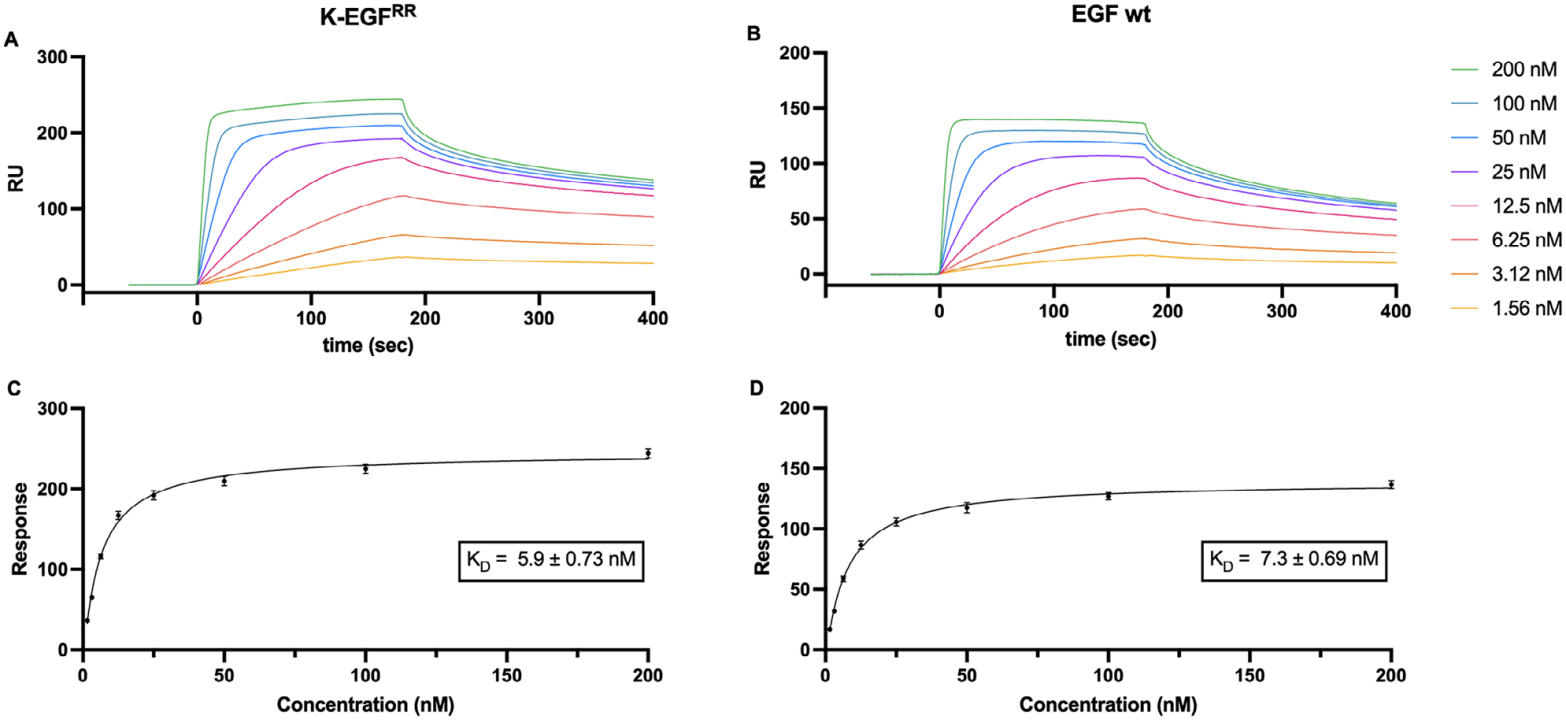
Higher ligand–receptor affinity in the case of K‐EGF^RR^ compared to commercially available wild‐type EGF determined by SPR. Biotinylated EGFR was immobilized on a streptavidin‐coated SPR chip and treated with different amounts of (A) K‐EGF^RR^ or (B) commercially available EGF. (C, D) The equilibrium dissociation constant *K*_D_ was determined by using the steady‐state affinity fit. The mean value ± SEM is shown. (*N* = 1, *n* = 2).

To gain a comprehensive understanding of the binding affinity between the K‐EGF^RR^ variant and the EGFR, we conducted two complementary sets of experiments: ELISA (Figure 4) and SPR (Figure 5). In the ELISA, we immobilized the biotinylated EGF receptor onto streptavidin‐coated well plates. We incubated the wells with PBS, the ligand variant K‐EGF^RR^, or the wild‐type EGF. Notably, the absorbance measured at 450 nm for K‐EGF^RR^ was even higher than that observed for the wild‐type EGF (Figure 4), suggesting a higher degree of binding to the EGFR.

Next, we measured the affinity of the ligand–receptor interaction by SPR. We immobilized the biotinylated receptor EGFR on a SPR sensor chip coated with streptavidin. The resulting curves for EGF binding demonstrated a moderate to fast association, leading to an equilibrium state followed by dissociation. Using the steady‐state affinity fit, the equilibrium dissociation constant *K*_D_ was calculated. The *K*_D_ of K‐EGF^RR^ was comparable to that of the commercially available EGF (5.9 × 10^−9^ vs. 7.3 × 10^−9^ M) (Figure 5). This similarity suggests that the variant and the wild type interact with the EGFR in an analogous way, which strongly supports the ELISA result. The data demonstrate that K‐EGF^RR^ preserves the EGFR binding properties of wild‐type EGF, displaying a high‐affinity binding in the low nanomolar range.

### 3.5 Specific Receptor‐Mediated Endocytosis of K‐EGF^RR^ in Cancer Cells

**FIGURE 6:**
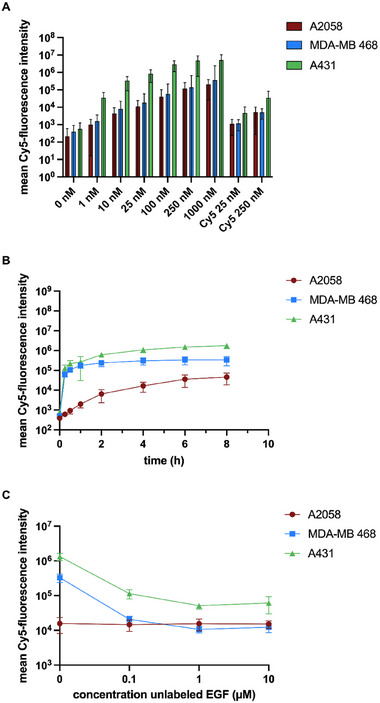
Receptor‐mediated uptake of the Cy5‐labeled EGF variant determined by flow cytometry. K‐EGF^RR^ was evaluated in cell lines A2058 (EGFR–), MDA‐MB 468 (EGFR+) and A431 (EGFR++) (0.25 Cy5/EGF). (A) Concentration and (B) time‐dependent uptake of Cy5‐labeled K‐EGF^RR^ incubated for 24 h. (C) Competitive inhibition of the internalization of Cy5‐labeled K‐EGF^RR^ by unlabeled K‐EGF^RR^ in varying concentrations. The mean value ± SEM is shown (*N* = 3, *n* = 2).

Drug side effects are often caused by low drug specificity that results in off‐target action. To assess the specificity of interaction between K‐EGF^RR^ and target or off‐target cells, we used cancer cell lines naturally expressing varying levels of EGFR. Two were EGFR‐positive, the breast carcinoma MDA‐MB 468 and epidermoid carcinoma A431 cell lines, and one was EGFR‐negative, the melanoma cell line A2058 [38]. Cells were treated with increasing concentrations of Cy5‐labeled K‐EGF^RR^ and for different time intervals. The levels of Cy5‐labeled K‐EGF^RR^ associated with different cell lines were measured by flow cytometry. We observed a time‐ and concentration‐dependent uptake into the target (A431, MDA‐MB 468) and off‐target (A2058) cell lines (Figure 6A,B). Notably, the intensity of the Cy5‐fluorescence signal was significantly higher in EGFR‐positive cells compared to EGFR‐negative cells correlating with their known expression of the EGFR [38]. To determine whether the cell‐ligand interaction was indeed a result of ligand–receptor interaction, we conducted a competitive inhibition assay. In this assay, we simultaneously incubated cells with 100 nM Cy5‐K‐EGF^RR^ and varying concentrations of unlabeled EGF for 4 h. A reduction in the fluorescence intensity was observed for the EGFR‐positive cells as the concentration of the competitive nonlabeled EGF increased, with no effect on EGFR‐negative A2058 cells (Figure 6C), which suggests that the Cy5‐K‐EGF^RR^ uptake was receptor‐dependent for EGFR‐positive cells.

**FIGURE 7:**
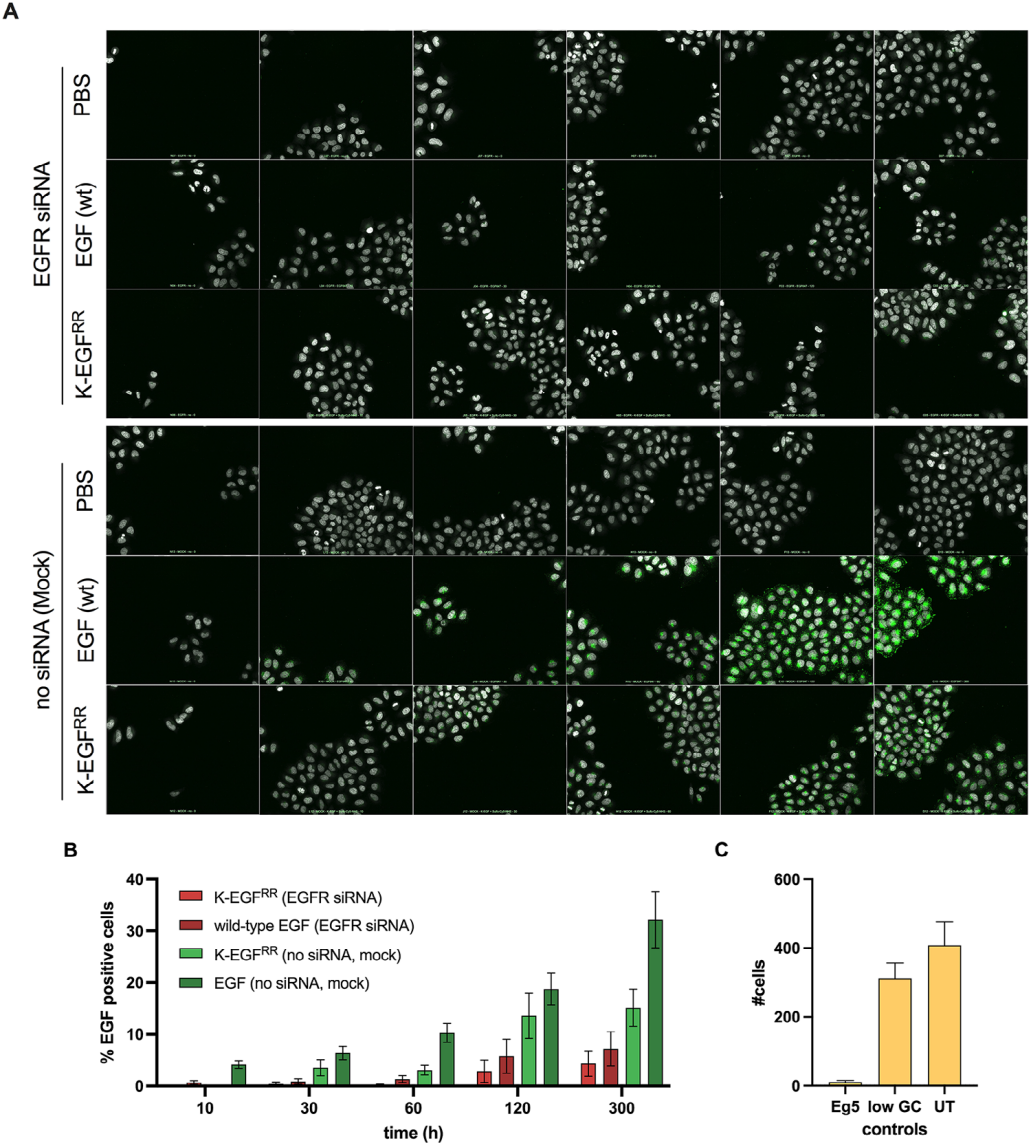
Receptor‐mediated endocytosis of EGF wild‐type and variant. (A) High‐throughput imaging of HeLa Kyoto cells either transfected with mock or siRNA against EGFR. Cells were treated with commercial EGF‐labeled with Alexa‐647 [EGF (wt), 9 nM], (sulfo‐Cy5‐NHS)0.25‐K‐EGF^RR^ [K‐EGF^RR^, 9 nM] or left untreated [DMEM] for 0, 10, 30, 60, 120 and 300 min (green). Cells were stained with a mix of DAPI and CellMask (grey). (B) Plot of the percentage of EGF‐positive cells for EGF (wt) and K‐EGF^RR^ in relation to overall cells for different time points and on cells treated with EGFR siRNA and mock. (C) Plot of the mean number of overall cells treated with different controls. The mean value ± SEM is shown (*N* = 4, *n* = 4).

To visualize receptor‐mediated internalization of Cy5‐K‐EGF^RR^ in cells, we performed uptake experiments using wild‐type and EGFR‐knocked‐down cervical carcinoma HeLa Kyoto cells. To this end, cells were treated using Eg5 siRNA as transfection control, EGFR siRNA to downregulate EGFR expression, and untargeted low GC siRNA as a negative control or no siRNA (mock). Subsequently, the cells were treated with 9 nM commercial wild‐type EGF labeled with Alexa‐647 or K‐EGF^RR^ labeled with 0.25 Sulfo‐Cy5‐NHS per ligand, while control cells were treated with PBS. Cells were then fixed and stained with a mixture of the DNA dye, DAPI, and the cytoplasmic dye, CellMask Blue (Figure 7A, grey), allowing individual cell segmentation. The internalization process was measured by following the fluorescently labeled EGF (Figure 7A, green, Figure S2) using automated high‐content imaging and quantitative image analysis.

First, we quantified the number of cells and estimated a transfection efficiency of 99% based on Eg5 siRNA viability (Figure 7C). Then, to determine if K‐EGF^RR^ is efficiently endocytosed by cells using receptor‐mediated endocytosis, we measured the number of EGF‐positive vesicles per cell in control and EGFR knockdown cells. Strikingly, we observed a dramatic decrease in K‐EGF^RR^‐positive vesicles upon EGFR knockdown, demonstrating that the EGF variant is specifically internalized by EGFR‐mediated endocytosis (Figure 7A,B, Figures S2 and S3). It is important to note that the fluorescence intensity of the wild‐type EGF was higher when compared to the mutant EGF. However, this can be explained because only 0.25 K‐EGF^RR^ ligands were labeled with the dye instead of 2–3 Alexa‐647 per wild‐type EGF. Nevertheless, the observed internalization kinetics are independent of the intensity of the staining. Our quantitative flow cytometry and unbiased image analyses comprising EGF competitive inhibition and EGFR knockdown experiments demonstrate the specific uptake of K‐EGF^RR^ by receptor‐mediated endocytosis in multiple EGFR‐positive cancer cell lines, making it an attractive candidate for targeted drug delivery.

## 4 Discussion

In our approach, we successfully achieved the recombinant expression of an active and soluble EGF variant using ArcticExpress(DE3) (AE(DE3)) or Rosetta(DE3) *E. coli* strain. The AE(DE3) strain allowed the protein to be expressed at a controlled rate and at a lower temperature (16°C), which minimized the risk of misfolding. Notably, when using Rosetta(DE3) instead of AE(DE3), the yield was increased from 250 µg/L to 5–6 mg/L. This is consistent with findings by Tegel et al., who compared protein yields of expressions in BL21(DE3) and Rosetta(DE3) [39]. However, when expressed in Rosetta(DE3), K‐EGF^RR^ was found to be encapsulated in inclusion bodies, which primarily comprise aggregates of overexpressed foreign proteins and often maintain a native‐like secondary structure [40, 41]. This secondary structure is reported to be advantageous during refolding and recovery of the expression product [42]. To recover the protein from the inclusion bodies we followed the procedure of Patra et al. [35] which involved the usage of 2 M urea for solubilization at pH 12. These conditions may be favorable as they reduce hydrophobic interactions [43]. However, it is worth noting that the recovery from inclusion bodies can sometimes result in misfolding and non‐active proteins. In our case, this was not observed, as confirmed by the binding studies of the EGF variant with EGFR‐positive cells and EGFR itself.

In fact, the actual receptor‐ligand interaction was evaluated by ELISA and SPR (Figures 4 and 5). Here, we observed that the K‐EGF^RR^ exhibits a binding affinity that is slightly higher or comparable to that of the wild‐type EGF. These results were confirmed in a cellular context, where we showed that K‐EGF^RR^ exhibits the ability to interact with cells by specific binding to the EGFR. This was supported by flow cytometry data (Figure 4) indicating that the binding and uptake of the Cy5‐labeled EGF variant was notably stronger in cells with an overexpression of EGFR, particularly in A431 cells. In addition, receptor‐mediated binding was confirmed by a competitive inhibition assay, which strongly suggests that internalization occurs due to receptor interaction. Moreover, when silencing EGFR with siRNA, the number of EGF‐positive cells and vesicles was significantly reduced, as quantified from unbiased high‐content images (Figure 7). This finding confirms that the variant's biological activity remains unaffected despite the substitution of lysines with arginines, as well as the introduction of an artificial lysine and His‐tag. Samples stored at –20°C were fully active even after 1 year. As the production of wild‐type EGF has been successfully upscaled to 5 L [33, 34], we assume that the upscaling process can be transferred to K‐EGF^RR^. For larger volumes such as 50 L, individual upscaling methods will probably have to be used, as has been established for other proteins [44].

Notably, in previous studies within our group the two lysines were replaced by arginines (RR) and the EGF variant was expressed as a fusion protein containing the toxin saporin. This mutation did not affect the EGF's ability to bind to EGFR nor the internalization of the saporin fusion protein [45]. In another study, it was shown that such an EGF^RR^ variant (without additional lysine) results in lower ERK phosphorylation than the wild‐type [30]. In other former studies, the wild type EGF was also recombinantly expressed as a fusion protein containing one of the toxins dianthin or saporin [46, 47]. This genetic fusion approach served the dual purpose of avoiding heterogeneity and crosslinking during chemical conjugation but is—in contrast to click‐K‐EGF^RR^—not applicable to nonprotein payloads such as siRNA or small molecular mass drugs.

As previously mentioned, for natural ligands such as EGF, potentially multiple conjugation sites of amino acids are often replaced by other amino acids to create a single conjugation site. For this purpose, Levashova et al. [48] modified the wild‐type EGF by adding an artificial cysteine to the N‐terminus for DOTA conjugation, which however requires that the existing disulfide bridges remain unmodified. Our deliberate modification, which is comprised of a well‐defined single conjugation site, enables us to attach the EGF variant to various effector or carrier systems. As a proof of concept, we employed heterobifunctional PEG linkers. These linkers featured an NHS ester on one end, facilitating the reaction with the single lysine and a DBCO group on the other hand, allowing an orthogonal, fast, and highly efficient click‐reaction, namely SPAAC, with a reaction partner possessing an azido group. The choice of the DBCO group offered advantages over the conventional copper‐catalyzed azide‐alkyne cycloaddition, particularly in drug delivery applications, as it does not rely on copper catalysts due to the ring strain of DBCO [49]. As previously mentioned, the employed linker can be either non‐cleavable or cleavable under reducing conditions. Cleavable linkers offer the distinct advantage of releasing the cargo within target organelles, ensuring that the drug's activity remains unaffected by the ligand.

## 5 Concluding Remarks

Through a series of experiments, we demonstrated that K‐EGF^RR^ exhibited comparable or even superior binding affinity to wild‐type EGF. Furthermore, we expanded its versatility by enabling the attachment of a click‐adapter to this single conjugation site. This strategic design positions K‐EGF^RR^ as an ideal candidate for targeted drug delivery, offering a ready‐to‐use platform that seamlessly links with various payloads using biorthogonal click‐chemistry.

## Conflicts of Interest

The authors have declared no conflicts of interest.

## Supporting information



Supporting Information

Supporting Information

Supporting Information

Supporting Information

## Data Availability

The data that support the findings of this study are available from the corresponding author upon reasonable request.
