# Supplementary material for: Recombinant Expression of a Ready‐to‐Use EGF Variant Equipped With a Single Conjugation Site for Click‐Chemistry
Source: Eng Life Sci. 2025 Mar 17;25(3):e70015. doi: 10.1002/elsc.70015 (PMC11913717; doi:10.1002/elsc.70015)
Supplement: Supplementary file 1 — Supporting Information [file ELSC-25-e70015-s001.docx]

**Figure legends for supplementary figures**

*Figure S1: Sequence data of K-EGF^RR^.*

(A) DNA sequence with start and stop codon printed in bold. The sequence includes initial and terminal restriction sites *Nco*I and *Eco*RI. (B) Protein sequence with the additional lysine and arginine mutations in bold.

*Figure S2: Receptor-mediated endocytosis of EGF wild-type and variant.*

(A) High-resolution imaging of HeLa Kyoto cells either transfected with siRNA against EGFR (A, B) or mock (C, D). Cells were treated with commercial EGF (A, C) labelled with Alexa-647 [EGF (wt), 9 nM] or (sulfo-Cy5-NHS)0.25-K-EGF^RR^ (B, D) [K-EGF^RR^, 9 nM] for 300 min (green). Cells were stained with a mix of DAPI and CellMask (grey).

*Figure S3: Receptor-mediated endocytosis of EGF wild-type and variant.*

Plot of the mean number of (A) overall cells and (B) cells positive for EGF (wt) and K-EGF^RR^ for different time points and on cells treated with EGFR siRNA and mock. The mean value ± SEM is shown (N = 4, n = 4).
