# Supplementary material for: Recombinant Expression of a Ready‐to‐Use EGF Variant Equipped With a Single Conjugation Site for Click‐Chemistry
Source: Eng Life Sci. 2025 Mar 17;25(3):e70015. doi: 10.1002/elsc.70015 (PMC11913717; doi:10.1002/elsc.70015)
Supplement: Supplementary file 2 — Supporting Information [file ELSC-25-e70015-s003.docx]

**A**

CC**ATG**GGCAAAGGAAATAGTGACTCTGAATGTCCCCTGTCCCACGATGGGTACTGCCTCCATGATGGTGTGTGCATGTATATTGAAGCATTGGACCGTTATGCATGCAACTGTGTTGTTGGCTACATCGGGGAGCGATGTCAGTACCGAGACCTGCGCTGGTGGGAACTGCGCGGTGGCCATCATCATCATCATCAT**TAA**GAATTC

**B**

MG**K**GNSDSECPLSHDGYCLHDGVCMYIEALD**R**YACNCVVGYIGERCQYRDL**R**WWELRGGHHHHHH
