# Supplementary figures and images for: Recombinant Expression of a Ready‐to‐Use EGF Variant Equipped With a Single Conjugation Site for Click‐Chemistry
Source: Eng Life Sci. 2025 Mar 17;25(3):e70015. doi: 10.1002/elsc.70015 (PMC11913717; doi:10.1002/elsc.70015)

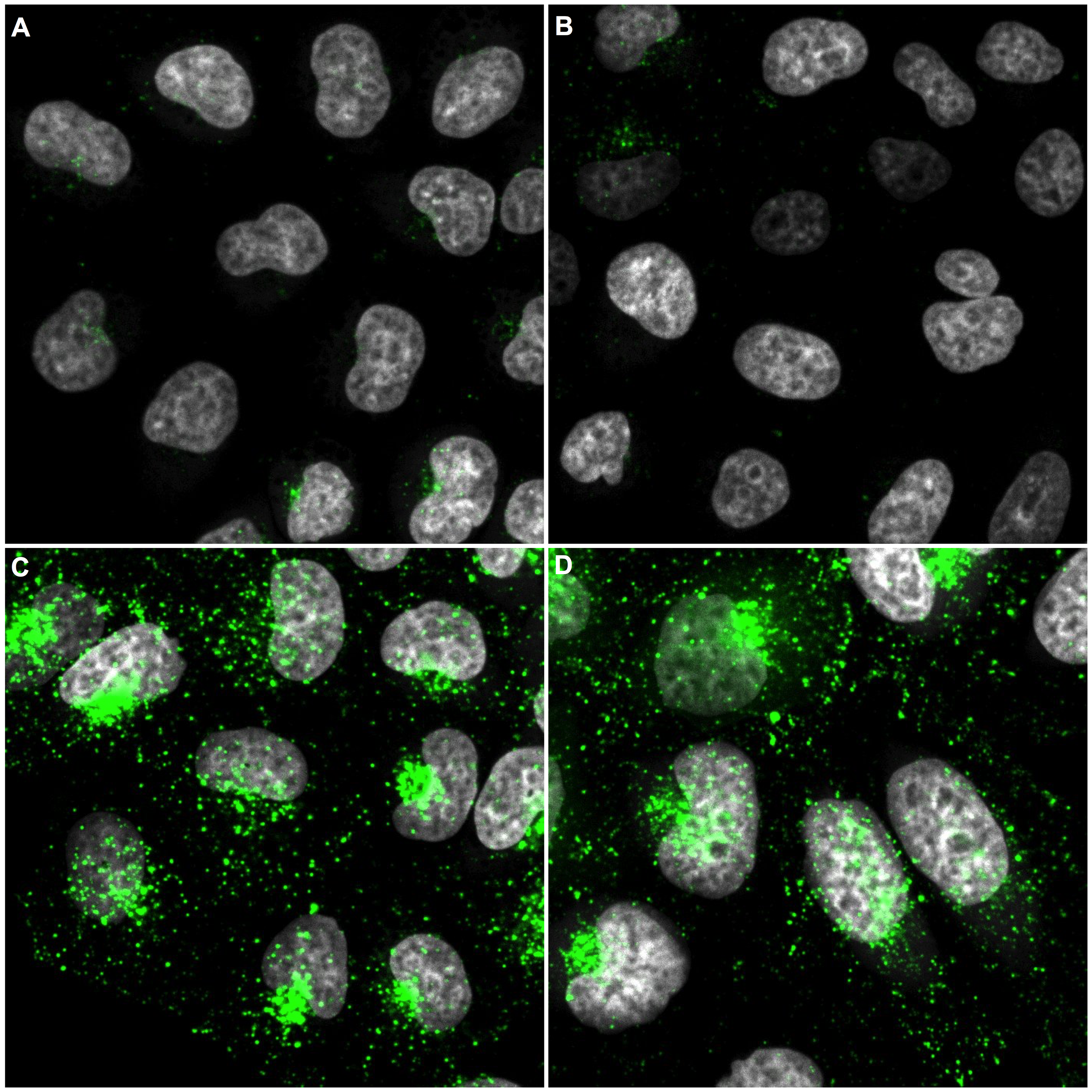

Supplement: Supplementary file 3 — Supporting Information [file ELSC-25-e70015-s004.tiff]

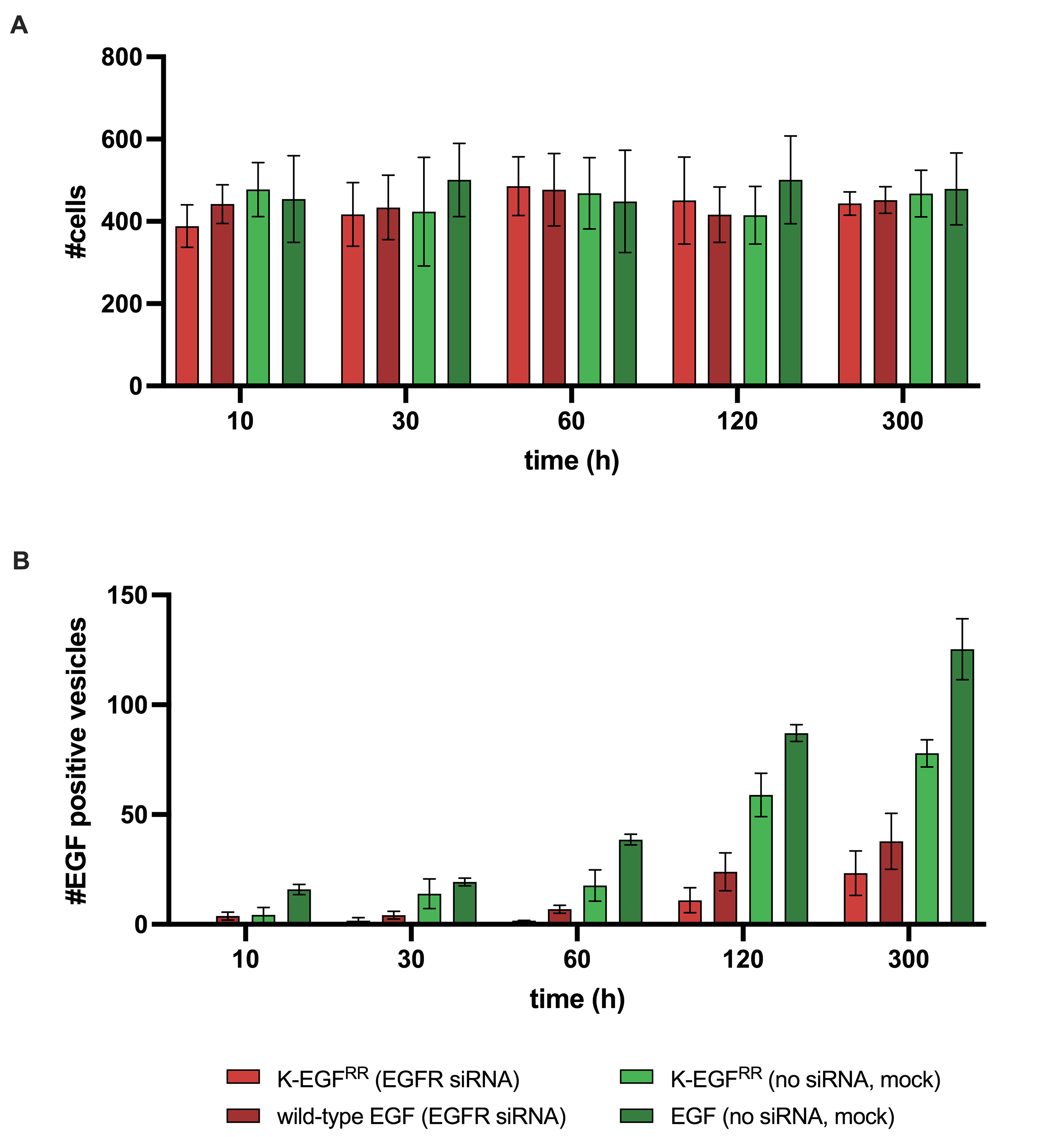

Supplement: Supplementary file 4 — Supporting Information [file ELSC-25-e70015-s002.tiff]
